# Imported *Hyalomma* ticks in the Netherlands 2018–2020

**DOI:** 10.1186/s13071-021-04738-x

**Published:** 2021-05-07

**Authors:** Mathilde Uiterwijk, Adolfo Ibáñez-Justicia, Bart van de Vossenberg, Frans Jacobs, Paul Overgaauw, Rolf Nijsse, Charlotte Dabekaussen, Arjan Stroo, Hein Sprong

**Affiliations:** 1 Centre for Monitoring of Vectors (CMV), National Reference Laboratory, Netherlands Food and Consumer Product Safety Authority (NVWA), Wageningen, the Netherlands; 2 National Plant Protection Organization (NPPO-NL), National Reference Laboratory, Netherlands Food and Consumer Product Safety Authority (NVWA), Wageningen, the Netherlands; 3 Institute for Risk Assessment Sciences (IRAS), Faculty of Veterinary Medicine, Utrecht University, Utrecht, the Netherlands; 4 Department of Infectious Diseases and Immunology, Faculty of Veterinary Medicine, Utrecht University, Utrecht, the Netherlands; 5 Centre for Infectious Disease Control (CIb), National Institute for Public Health and the Environment (RIVM), Bilthoven, the Netherlands

**Keywords:** Surveillance, Vector-borne disease, One Health, Viral haemorrhagic fever, Cluster analysis, Citizen science

## Abstract

**Background:**

Ticks of the genus *Hyalomma*, which are vectors for several tick-borne diseases, are occasionally found in areas outside their endemic range including northern parts of Europe. The objective of this study was to analyse adult *Hyalomma* ticks that were recently found in the Netherlands.

**Methods:**

*Hyalomma* ticks were morphologically identified. Cluster analysis, based upon sequence data (*cox1* barcoding) for molecular identification, and pathogen detection were performed. Additionally, a cross-sectional survey of horses was conducted to actively search for *Hyalomma* ticks in summer 2019. Analysis of temperature was done to assess the possibility of (i) introduced engorged nymphs moulting to adults and (ii) establishment of populations in the Netherlands.

**Results:**

Seventeen adult *Hyalomma* ticks (one in 2018, eleven in 2019, five in 2020) were found by citizens and reported. Fifteen ticks were detected on horses and two on humans. Twelve were identified as *H. marginatum*, one as *H. rufipes* and four, of which only photographic images were available, as *Hyalomma* sp. No Crimean-Congo haemorrhagic fever virus or *Babesia*/*Theileria* parasites were detected. One adult tick tested positive for *Rickettsia aeschlimannii*. In the cross-sectional horse survey, no *Hyalomma* ticks were found. Analysis of temperatures showed that engorged nymphs arriving on migratory birds in spring were able to moult to adults in 2019 and 2020, and that cumulative daily temperatures in the Netherlands were lower than in areas with established *H. marginatum* populations.

**Conclusions:**

Our results show that *Hyalomma* ticks are regularly introduced in the Netherlands as nymphs. Under the Dutch weather conditions, these nymphs are able to develop to the adult stage, which can be sighted by vigilant citizens. Only one human pathogen, *Rickettsia aeschlimannii*, was found in one of the ticks. The risk of introduction of tick-borne diseases via *Hyalomma* ticks on migratory birds is considered to be low. Establishment of permanent *Hyalomma* populations is considered unlikely under the current Dutch climatic conditions.
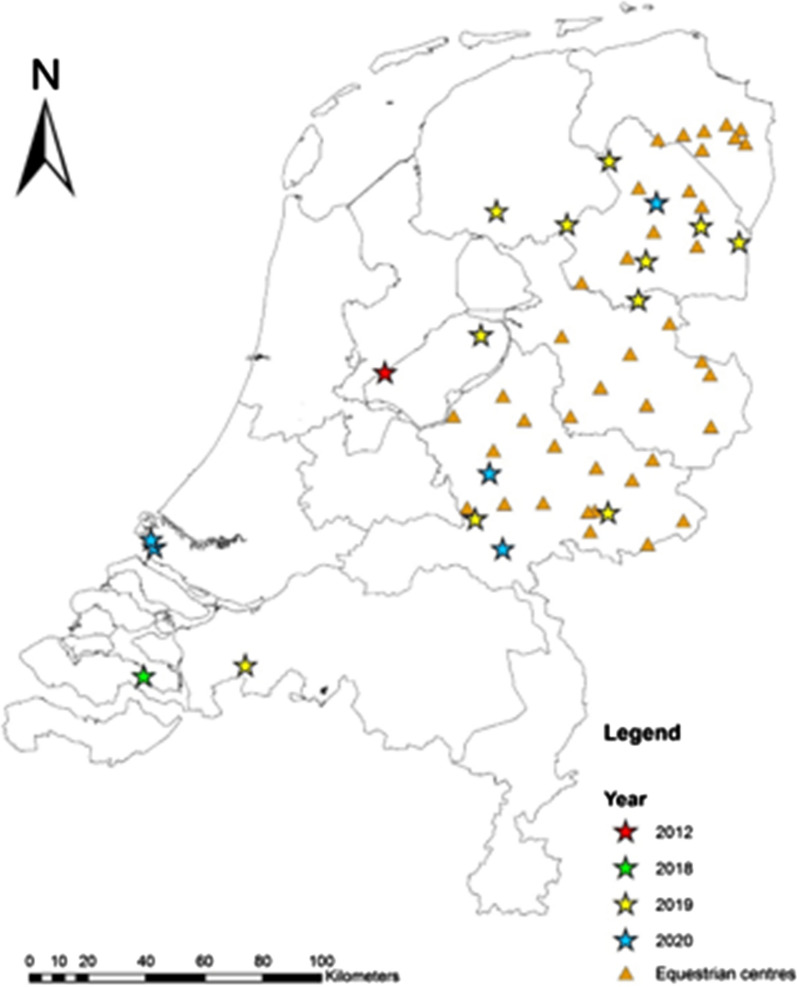

**Supplementary Information:**

The online version contains supplementary material available at 10.1186/s13071-021-04738-x.

## Background

Ticks of the genus *Hyalomma* (Acari: Ixodidae) are endemic in semi-arid regions across Asia, Africa and Europe [1–3]. Incidentally, specimens of *Hyalomma marginatum* sensu lato (Koch, 1844) have been reported far from the boundaries of their endemic range [4]. Migrating birds play an important role in long-distance dispersal of immature stages of *H. marginatum* s.l. into new areas. In studies performed in southern and eastern parts of Europe for which thousands of migratory birds were checked for ticks, several hundreds of larvae and nymphs of *Hyalomma* spp. were collected [5–14]. In northern and western parts of Europe such as in the United Kingdom [11], Sweden [12], Norway [13] and the Netherlands [15], immature stages of *Hyalomma* spp. were also found on migrating birds, but to a lesser extent. Occasionally, adults of *H. marginatum* have been found in northern and western European countries [16–23]. In the Netherlands, three adult *Hyalomma* sp. ticks were reported on horses between 2005 and 2009 [24, 25], and one adult *Hyalomma* sp. tick was found attached to a person in 2012 [26].

*Hyalomma marginatum* is a species complex including *H. marginatum*, *H. turanicum* and *H. rufipes* [27]. *Hyalomma marginatum* are two-host ticks. The larvae and nymphs feed on small mammals, ground-dwelling birds and reptiles [28]. Larval feeding, moulting to nymphal stage and nymphal feeding will in most cases occur on the same host, on which the immature stages can be present for up to 26 days [29, 30]. Adult *Hyalomma* ticks feed on ungulates such as Bovidae, Equidae, Cervidae and Suidae, but also (smaller) mammals such as Canidae, Lagomorpha and occasionally humans [28, 31].

*H. marginatum* ticks can transmit tick-borne pathogens such as Crimean–Congo haemorrhagic fever virus (CCHFv) and spotted fever rickettsiae to humans, *Babesia caballi* and *Theileria equi* (piroplasmosis) to horses, and *T. annulata* (tropical theileriosis) to bovines. Therefore, this tick species is of public and veterinary health concern [28, 32–34].

In this study we describe citizen reports of adult *Hyalomma* ticks in the Netherlands, and the results of a survey that was undertaken to actively search for *Hyalomma* ticks after the first sightings in 2019. The results of the molecular identification and the pathogen analysis of the specimens are discussed. Furthermore, we evaluate the possibility of nymph moulting, wintering and establishment of *Hyalomma* ticks after introduction in the Netherlands, based upon climatic data.

## Methods

### Tick collection

#### Citizens’ notification

From the beginning of summer 2019, and after receiving the first notification of *Hyalomma,* citizens were engaged to report *Hyalomma* ticks by posting information on the websites of the Netherlands Food and Consumer Product Safety Authority (NVWA) (www.nvwa.nl/reuzenteek) and the National Institute for Public Health and the Environment (RIVM) (www.rivm.nl/tekenbeten-en-lyme/hyalomma-teken). Citizens could send photographic images of *Hyalomma* ticks to the Centre for Monitoring of Vectors (CMV, NVWA, National Reference Centre, the Netherlands) via email or using the specific *Hyalomma* tick report system included on the NVWA website. After evaluation of the photographic images by entomologists, suspected *Hyalomma* specimens were transported to the laboratory and the contributors were questioned for additional information by telephone or on-site. When on-site, relevant animals present and vegetation nearby were carefully inspected for other possible *Hyalomma* ticks.

#### Cross-sectional horse study

**Fig. 1:**
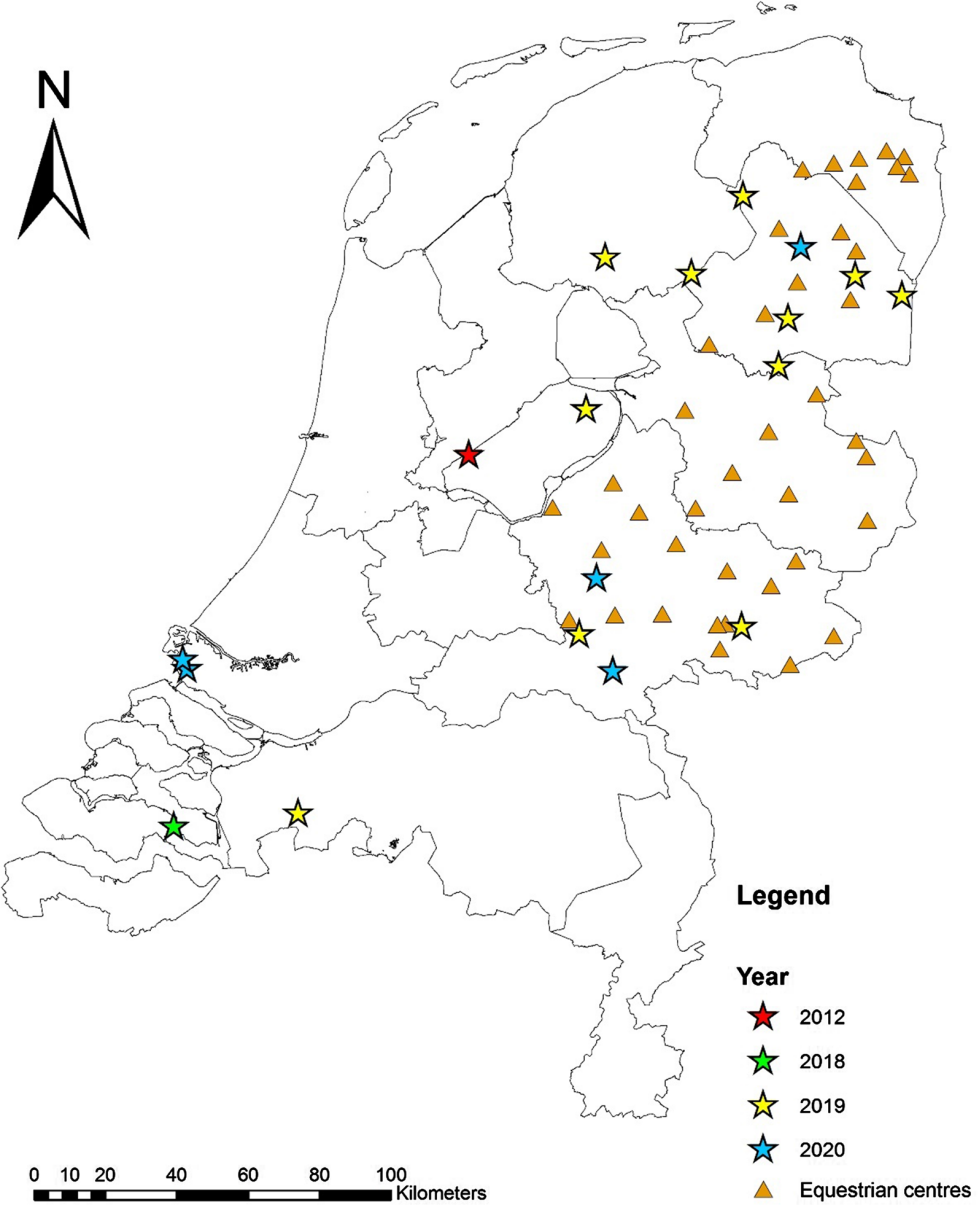
Location of *Hyalomma* ticks found in the Netherlands (stars), and equestrian centres visited for the horse survey in 2019 (triangles)

After receiving the first notification of *Hyalomma*, a cross-sectional study was performed to investigate the presence of *Hyalomma* ticks on horses. For this, 40 horse farms and equestrian centres, located in rural wooded areas in the northern and eastern part of the Netherlands, were visited between August and October 2019 (Fig. 1). Locations were visited once, and at each location five horses were carefully examined for ticks, in particular the nose, neck, chest, under the manes and tail, between the legs and the legs themselves. Horses were selected based upon having outdoor activities on a regular basis and/or being grazed and/or frequently being infested with ticks in the past.

### Tick identification

#### Morphological identification

Ticks that arrived in the laboratory (Table 1) were morphologically identified by tick specialists according to the keys of Estrada-Peña et al. and Walker et al. [35, 36]. After morphological identification, the ticks were immediately processed for molecular and pathogen detection or first individually stored at −80 °C before further analysis.

**Table 1:** Information about *Hyalomma* ticks and results of laboratory analyses (morphological and molecular identification, and pathogen detection)

Date found	Municipality name	Province	Found on	Stage	M/F	Morphological identification	Cluster analysis	Genbank acc. no.	CCHFv	*Rickettsia*	*Theileria*/*Babesia*
5 June 2012	Almere	Flevoland	Bird	Nymph	n.a.	*Hyalomma* sp.	*H. rufipes*	MT757612	−	−	−
18 September 2018	Yerseke	Zeeland	Horse	Adult	M	*Hyalomma* sp.*	x	x	x	x	x
4 July 2019	Zelhem	Gelderland	Horse	Adult	F	*Hyalomma* sp.*	x	x	x	x	x
14 July 2019	Odoorn	Drenthe	Horse	Adult	F	*H. marginatum*	*H. marginatum*	MT757622	−	+	−
30 July 2019	Dronten	Flevoland	Horse	Adult	F	*H. marginatum*	*H. marginatum*	MT757619	−	−	−
6 August 2019	Oldeouwer	Friesland	Human	Adult	M	*Hyalomma* sp.*	x	x	x	x	x
7 August 2019	Emmer-Compascuum	Drenthe	Horse	Adult	M	*Hyalomma* sp.	*H. marginatum*	MT757618	−	−	−
14 August 2019	Linde	Drenthe	Horse	Adult	F	*H. marginatum*	*H. marginatum*	MT757613	−	−	−
14 August 2019	Eén	Drenthe	Horse	Adult	F	*H. marginatum*	*H. marginatum*	MT757621	−	−	−
20 August 2019	Tiendeveen	Drenthe	Horse	Adult	M	*H. marginatum*	*H. marginatum*	MT757616	−	−	−
23 August 2019	Noordwolde	Friesland	Horse	Adult	F	*H. marginatum*	*H. marginatum*	MT757615	−	−	−
27 August 2019	Rucphen	Noord-Brabant	Human	Adult	M	*H. marginatum*	*H. marginatum*	MT757614	−	−	−
20 September 2019	Wageningen	Gelderland	Horse	Adult	F	*H. marginatum*	*H. marginatum*	MT757617	−	−	−
4 May 2020	Amen	Drenthe	Horse	Adult	F	*Hyalomma* sp.	*H. marginatum*	MT757620	−	−	−
4 August 2020	Oosterhout	Gelderland	Horse	Adult	F	*Hyalomma* sp.	*H. marginatum*	MW495246	−	−	−
11 August 2020	Rockanje	Zuid-Holland	Horse	Adult	F	*H. rufipes*	*H. rufipes*	MW495248	−	−	−
15 August 2020	Oostvoorne	Zuid-Holland	Horse	Adult	F	*Hyalomma* sp.	*H. marginatum*	MW495247	−	−	n.t.
4 September 2020	Harskamp	Gelderland	Horse	Adult	Unknown	*Hyalomma* sp.*	x	x	x	x	x

+  detected, − not detected, * identification based on photographic images, *x* specimen not available for laboratory analyses

*M* male, *F* female, *CCHFv* Crimean–Congo haemorrhagic fever virus, *n.a.* not applicable, *n.t.* not tested

#### Molecular identification and cluster analysis

Molecular identification was performed by Sanger sequencing of the partial mitochondrial *cox1* gene using primers LCO1490 and HCO2198 [37] according to the European and Mediterranean Plant Protection Organization (EPPO) DNA barcoding standard PM7/129(1) [38]. In short, a few legs of the specimen were used as input for the DNA extraction. Tick tissue was ground in a lysis buffer with a micro-pestle prior to DNA extraction with the High Pure^®^ polymerase chain reaction (PCR) template preparation kit (Roche, Basel, Switzerland). Positive and negative controls were always included to detect possible contamination and to monitor the efficiency of Sanger sequencing and assembly. Amplicons were purified with the QIAquick^®^ PCR Purification Kit (Qiagen, Venlo, the Netherlands) prior to cycle sequencing with both amplification primers in individual runs using the BigDye^®^ Terminator v1.1 Cycle Sequencing Kit (Life Technologies, Carlsbad, CA). Cycle sequence products were purified with DyeEx^®^ 2.0 Spin Kit columns (Qiagen, Venlo,  the Netherlands) prior to sequencing on a 3500 Genetic Analyzer (Life Technologies, Carlsbad, CA).

Electropherograms were assembled in Geneious Prime 2019.2.3 (Biomatters, Auckland, New Zealand), and consensus sequences were aligned with selected NCBI accessions using MAFFT [39]. After masking 5′ and 3′ overhang sequences in the 896-base-pair (bp) alignment, 542 bp were represented by all specimens in the alignment which were included in the clustering analysis. The alignment was subjected to a UPGMA (unweighted pair group method with arithmetic mean) clustering analysis using the tree builder tool incorporated in Geneious Prime (HKY85 substitution model; 100 bootstraps).

*Hyalomma* sequences generated in this study were deposited in the NCBI GenBank^®^ database under the accession numbers MT757612 to MT757622, and MW495246 to MW495248.

### Pathogen detection

#### DNA and RNA extraction

For pathogen detection, the remaining tick bodies were lysed and homogenized in RNAlater^®^ (Thermo Fisher Scientific, Leiden, the Netherlands), and subsequently processed according to the manufacturer’s instructions in a diagnostic laboratory setting (Nucleic Acid Isolation Kit I; Roche). Extraction of whole nucleic acid from the ticks was performed using robot extraction (MagNA Pure Compact Extraction Robot; Roche, Basel, Switzerland). To detect potential cross-contamination, negative controls were included in each extraction batch. Samples were analysed with different (real-time) PCRs based on various genes specific for the microorganism of interest and carried out on a LightCycler^®^ 480 (Roche Diagnostics Nederland B.V., Almere, the Netherlands). Positive (plasmid) controls and negative water controls were used on every plate tested. To minimize contamination leading to false-positive samples, the DNA/RNA extraction, PCR mix preparation, sample addition and (q)PCR analyses were performed in separated air-locked dedicated labs.

#### Crimean–Congo haemorrhagic fever virus

For detection of CCHFv, a reverse transcription real-time PCR was performed [40].

#### *Babesia* and *Theileria*

Primers recognizing a 400–440-bp fragment of the 18S rRNA gene of both *Babesia* spp. and the closely related *Theileria* spp. were used for PCR amplification [41].

#### Rickettsiae

Lastly, for the detection and identification of spotted fever rickettsiae, real-time PCR was performed on the citrate synthase (*gltA*) gene [42]. On positive samples, the partial outer membrane protein *ompB* gene was amplified with conventional PCR and used for sequencing [43]. The *Rickettsia* sequence generated in this study was deposited in the NCBI GenBank^®^ database under accession number MW498244.

### Evaluation of *Hyalomma* tick moulting and wintering after introduction

We assume that the *Hyalomma* ticks found in the Netherlands were introduced on migratory birds in early spring. These ticks were introduced as feeding nymphs, and after processing the blood meal they completed the moult to the adult stage in the environment. This last step is a temperature-dependent process. We evaluated the possibility of engorged nymphs successfully moulting to adults using two different criteria: (i) about 300 °C cumulative degrees above the developmental zero (14–16 °C) is necessary to complete the moult [44], and (ii) temperatures of  > 8 °C for 15 continuous days ensure the moulting of nymphs [45].

We also evaluated the possibility of *Hyalomma* wintering and establishment using the temperature-related limiting factor described by Gray et al. (2009), where temperatures between September and December are critical for wintering of *H. marginatum* ticks. As suggested by these authors, and using a threshold of 10 °C, we considered that areas with average cumulative temperatures of 800 °C between September and December would be suitable and areas below 400 °C would be unsuitable for the species [46].

Daily average temperature values were obtained from available data of the Royal Netherlands Meteorological Institute (KNMI). For the calculations, we downloaded the data for the weather stations “De Bilt” (situated centrally in the Netherlands) and “Heino” and “Hoogeveen” situated in the vicinity of most of the *Hyalomma* sighting locations (Fig. 1).

## Results

### Tick specimens

#### Citizens’ notifications

From July 2019 to December 2020, 118 sightings of ‘*Hyalomma* ticks’ were reported (Additional file 1: Table S1). Also, one adult *Hyalomma* sp. tick had been found in 2018 but was reported in 2020. Of these, 17 specimens (one in 2018, 11 in 2019, five in 2020, see Table 1) could be classified as adult *Hyalomma* sp. ticks based upon photographic images provided by the citizens. Figure 1 shows the locations were the ticks were found.

The 102 reports which were not *Hyalomma* ticks were identified as other tick species (*n* = 51), mostly of the genera *Ixodes* sp. and *Dermacentor* sp., but also hemipterans (*n* = 15), spiders (*n* = 10), louse flies (*n* = 6), other arthropods or unidentifiable.

Of all reported adult *Hyalomma* ticks, nearly all (15/17) were discovered on horses. Two ticks were discovered on humans, both not attached. The horses on which the ticks were found were all kept (partly) on pasture, and most horses were taken for outdoor rides on a regular basis. None of the horses or persons on which the adult ticks were found had (recently) been abroad. We enquired where the ticks were most likely acquired, and whether similar ticks were observed recently or earlier. In about half of the reports, we checked on-site whether other specimens were present on animals or in the vegetation. Consequently, no other *Hyalomma* ticks were reported or detected. On average, it took 1.9 days (range 0–4 days) for a found tick to arrive at the laboratory. Nearly all ticks were dead on arrival at the laboratory.

### Morphological and molecular species identification and pathogen detection

Thirteen of the 17 reported adult *Hyalomma* ticks and one nymph that was found on a migratory bird (Eurasian reed warbler *Acrocephalus scirpaceus*) in 2012 were available for further investigation (Table 1).

#### Morphological species identification

Morphological analysis in the laboratory identified eight of the adult *Hyalomma* ticks as *H. marginatum* and one as *H. rufipes* (Table 1). Four adult ticks and the nymph could not be morphologically identified to species level with certainty in the laboratory (Table 1).

#### Molecular species identification and cluster analysis

**Fig. 2:**
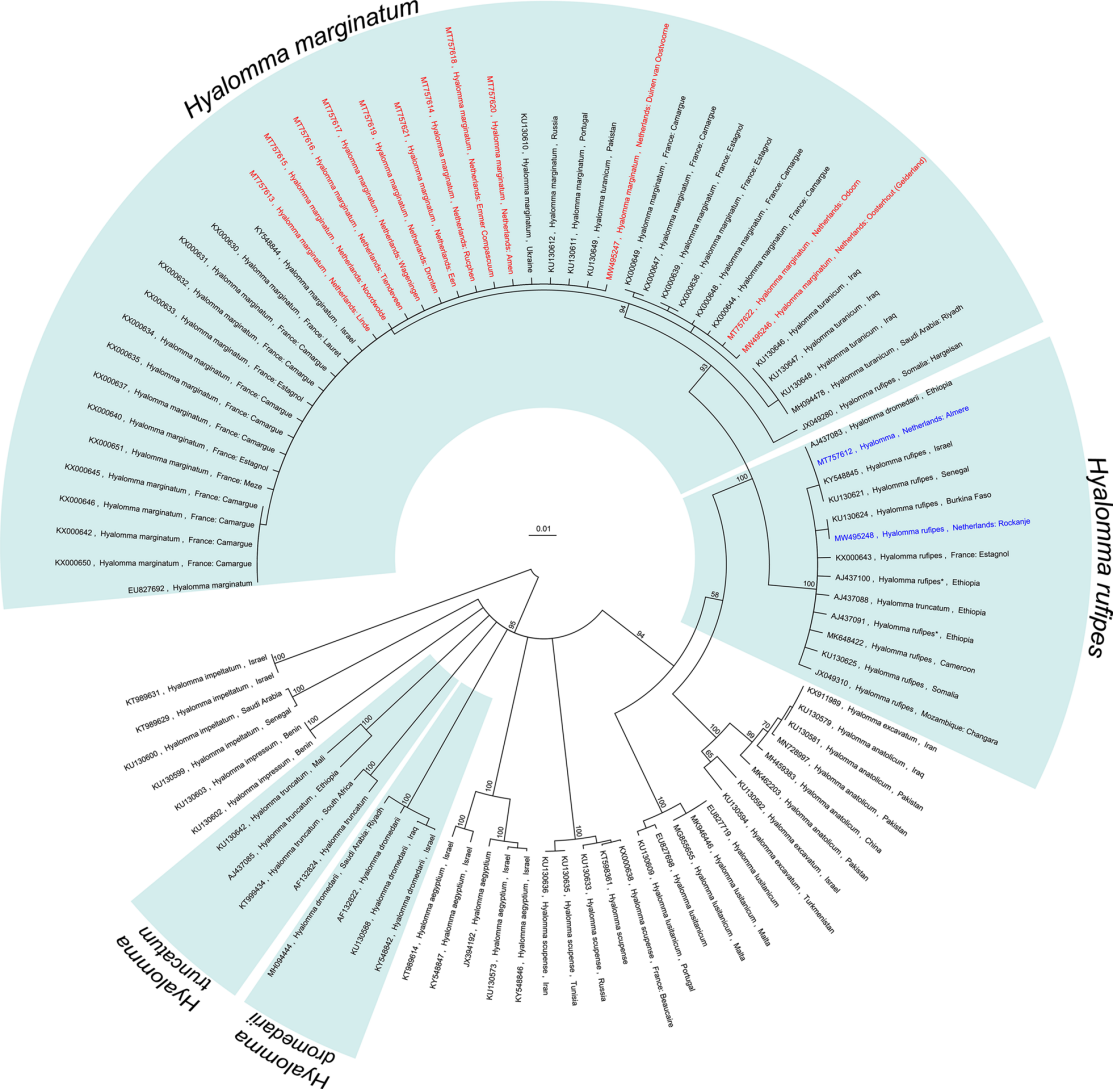
Cluster analysis of *Hyalomma* specimens. Cladogram of a fragment of the mitochondrial *cox1* gene based on 77 selected NCBI accessions and 12 *Hyalomma* specimens found during this study. Organism names of sequences indicated with an asterisk (*) have been altered relative to the NCBI record based on information provided in the original paper

A total of 91 partial *cox1* gene sequences were included in the alignment, representing 77 selected sequences deposited in NCBI GenBank^®^ (see Additional file 1: Table S2) and 14 specimens (13 adults and 1 nymph) from the Netherlands generated in this study. Results of the *cox1* sequence molecular analysis (UPGMA; HKY85, 100 bootstraps) showed that the specimens found in our study cluster in two major clades (Fig. 2). The first clade, with 98% bootstrap support, clusters 12 adult specimens with *H. marginatum* (24), *H. turanicum* (5) and *H. rufipes* (1) NCBI sequences. The second clade, with 100% bootstrap support, clusters one adult specimen and the nymph with *H. rufipes* (9), *H. dromedarii* (1) and *H. truncatum* (1) NCBI sequences. As shown in Fig. 2, *H. dromedarii* and *H. truncatum* sequences are also represented in other clades. According to combined morphological and molecular identification (Table 1), 12 adult ticks can be classified as *H. marginatum* and one as *H. rufipes*. The nymph can be classified as *H. rufipes*.

#### Pathogen detection

Results of the pathogen detection analysis (Table 1) show that one adult *H. marginatum* tick reported in 2019 tested positive for *Rickettsia* sp., more specifically *R. aeschlimannii*. In the other 13 *Hyalomma* ticks that were tested, no pathogens were detected.

### Cross-sectional horse study

A total of 202 horses from 40 equestrian centres (Fig. 1) were examined for the presence of ticks in 2019. In this survey, no *Hyalomma* ticks were found during the inspections or reported by the caretakers. Six *Ixodes* ticks were found on five horses.

### Accumulated daily temperature analysis

Using the parameters obtained in the laboratory by Emelyanova et al. (2005), and using the lowest developmental zero temperature suggested (*T*_0_ = 14 °C), if *Hyalomma* nymphs were introduced early in spring in 2019 (1st of April), results show that the first adult would be expected to appear approximately during the first days of August in 2019. This result does not match the first *Hyalomma* adult sighting in 2019 reported 1 month earlier (on 4th of July).

However, results applying the temperature parameters used in the study by Gale et al. (2012) show that in the spring of 2019 (from 15 April 2019 until 2 May 2019) and 2020 (from 15 April 2020 until 2 May 2020) temperatures of > 8 °C for 15 or more continuous days were recorded prior to the citizens' sightings of *Hyalomma* adults, allowing the engorged nymphs to moult to adults.

In 2018 and 2019, cumulative temperatures above 10 °C between September and December (weather station “ Heino”) totalled 216 °C and 192 °C, respectively, indicating that this area is not considered suitable for the establishment of populations of *H. marginatum* (cumulative temperature below 400 °C), according to Gray et al. [46].

## Discussion

Ticks of the *H. marginatum* complex are known vectors of diseases of veterinary and public health importance, and are able to survive in a wide range of climatic conditions and a variety of habitats [31]. In this study, we report citizens' notifications of *Hyalomma* adults in the Netherlands. Results of this study raise questions about the frequency of introductions and the ability to establish endemic *Hyalomma* populations in the Netherlands which calls for vigilance because of the possible risk to human and animal health.

Even though *Hyalomma* ticks have been found and reported before in the Netherlands [15, 24–26], the numbers of adult *Hyalomma* ticks reported within the past few years seem unprecedented. In the weeks preceding the sightings in 2019, the media reported about adult *Hyalomma* ticks in horse farms in Germany [47] in a sensationalistic way. The sighting of the first *Hyalomma* ticks in the Netherlands that year boosted the media attention. There, the *Hyalomma* ticks were described as ‘giant’, ‘monster’ or ‘horror’ ticks and were connected to ‘a deadly Ebola-like virus’ (meaning CCHFv), which probably led to an increased awareness among the public. Fifteen out of 17 adult *Hyalomma* ticks were discovered on horses. This is most likely explained by the fact that horses, in contrast to other hosts for *Hyalomma* adults, are closely inspected and handled on a regular basis by their caretakers during grooming and saddling.

Of the thirteen available adult *Hyalomma* ticks (Table 1), eight could be morphologically identified as *H. marginatum* and one as *H. rufipes.* Four could not be morphologically identified to species level with certainty, due to the poor state (dried out and/or moldy) of the specimens received. Also, the engorged nymph that was found on a migratory bird in 2012 [15] could not be morphologically identified to species level, because of the difficulties arising in morphological identification of *Hyalomma* nymphs to species level [27, 35].

Molecularly, 12 adults, including the eight ticks that were morphologically identified as *H. marginatum*, clustered mainly with *H. marginatum* (24). Also in this cluster were two other members of the *H. marginatum* species complex, *H. turanicum* (5) and *H. rufipes* (1) (Fig. 2). The one adult that was morphologically identified as *H. rufipes* and the nymph clustered mainly with *H. rufipes* (9). One *H. dromedarii* and one *H. truncatum* were also in this cluster, which also clustered together in their own cluster (Fig. 2). Ticks from the *H. marginatum* species complex are known to be taxonomically challenging to identify [27]. Also, cryptic hybridization in *Hyalomma* ticks might at least partly account for the apparent incongruence between morphology and molecular clustering [48].

*H. rufipes* has also been found in western and northern European countries [13, 18]. To our knowledge, *H. dromedarii* and *H. truncatum* specimens have not been found in western and northern European countries. In our consideration, we also took into account the fact that *H. rufipes* is a two-host tick of which the immatures are most likely to seek (migratory) birds as hosts, in contrast to *H. dromedarii* and *H. truncatum* [35, 36]. Combining morphological and molecular identification, we classified the 12 adults as *H. marginatum*, and both the adult and nymph as *H. rufipes*.

The ticks were most likely introduced via migratory birds. In general, migrating birds that breed in the Netherlands and winter in warmer climates can be divided into two groups. Birds belonging to the first group migrate over long distances, and winter in Africa (Sahel to more southern regions). The mass arrival in the Netherlands of these birds, such as garden warbler (*Sylvia borin*), common whitethroat (*S. communis*), willow warbler (*Phylloscopus trochilus*) and common redstart (*Phoenicurus phoenicurus*), is from mid-April to May. The second group consists of birds that winter much nearer, in southern Europe and northern Africa. These birds, such as the European robin (*Erithacus rubecula*), song thrush (*Turdus philomelos*), common chiffchaff (*Phylloscopus collybita*) and Eurasian blackcap (*Sylvia atricapilla*), arrive from March onwards, leaving the African and Mediterranean European countries already in February. In the wintering and stop-over areas of both groups, *Hyalomma* ticks are endemic and can be dispersed via returning migrating birds [49]. Indeed, *Hyalomma* immatures have also been found on bird species of both groups returning to or present in their breeding area [5–13, 50–52], also in the Netherlands [15]. The *Hyalomma* ticks reported in this study were most likely introduced at the locations in early spring by migratory birds as engorged nymphs, and moulted to adult stage. The horses and persons on which the adult ticks were found had not (recently) been abroad. Also, in case of introduction of adult ticks with animal hosts (e.g. introduced via imported horses), more specimens found at one location would be expected [31, 33, 46].

As shown by the results of the cumulative daily temperature analyses, the temperatures recorded between September and December of 2018 and 2019 do not correspond to the areas where *H. marginatum* has permanent populations. As suggested by Gray et al. (2009), these parameters (average cumulative temperature of 800 °C in places where the tick has permanent populations, and below 400 °C in sites not colonized) are related to local factors that affect the moulting of nymphs to adult stage before the onset of winter. These parameters are not related to (extreme) cold winter temperatures that prevent wintering adults from surviving into the next year, because unfed adults of *Hyalomma* are very capable of surviving even harsh winter conditions in continental climates, probably hidden deeply in the litter layer [32, 46]. Therefore, temperatures in late summer and autumn are more critical for potential *Hyalomma* survival and establishment of permanent populations than temperatures in winter.

Results according to the Emelyanova et al. criteria [44] show that the early sightings (before August) of adult *Hyalomma* ticks in the Netherlands do not align with the suggested temperature accumulation. According to these criteria, introduced engorged nymphs in 2019 and 2020 could not moult to adults within this time period, suggesting that adult *Hyalomma* ticks that were sighted before August wintered at these locations. A period of at least 4 months would have been necessary in 2019 for engorged nymphs to moult into adults. Taking into consideration the probability of survival of the moulting ticks during such a 4-month period exposed to (environmental) factors such as predation, parasitism, fungal infections, drowning or desiccation [31, 53, 54], this period can be considered too long and the proposed parameters less appropriate. Applying the criterion proposed by Gale et al. [45], engorged nymphs that arrived at the end of March or in April could have moulted to adults in the first days of May in 2019 and 2020, matching the sightings of the first adults. The criterion used by Gale et al. [45] and accepted for the *H. marginatum* populations maintained in Spain (Estrada-Peña personal communication) is based on temperature as the main factor affecting the seasonal pattern of the *H. marginatum* tick (Estrada-Peña et al. 2011) and is therefore used as the sole parameter for moulting. Other critical parts of the life cycle necessary for the establishment of permanent populations besides nymphal moulting and wintering of adults [46, 49, 53], such as oviposition by the adult females and questing and moulting activity of all stages, are heavily dependent on (micro)climatic conditions as well [53]. This explains why in areas where climatic conditions are favourable for moulting from nymph to adult, this does not automatically lead to permanent populations of *Hyalomma* ticks.

Besides climatic factors, tick densities and dispersal are also important factors for possible establishment of *Hyalomma* [28, 53]. All sightings of *Hyalomma* ticks so far were singular, and no other *Hyalomma* ticks were reported by the citizens or detected by us in the vicinity of the reported adult *Hyalomma* tick. Also, no *Hyalomma* ticks were found in the horse screening part of this study. Although it is likely that more *Hyalomma* ticks are present than sighted, we hypothesize that these ticks are still too dispersed to find a mating partner (Allee effect [55, 56]). With the current seemingly occasional introduction on migratory birds, the chance for adult females to encounter adult males for mating on the same mammalian host can be considered very low. However, we cannot conclude that this event is impossible or could not have already happened. To detect attached larvae or nymphs, active (more site-directed) monitoring on small mammals and resident birds would need to be implemented in early spring, at least in areas where *Hyalomma* has been sighted. Besides the above-mentioned factors, the availability and densities of suitable hosts are also important. Given that birds, rodents, lagomorphs, and wild and domestic ungulates are abundant in the Netherlands, this will probably not be the most limiting factor.

Taking the above-mentioned into account, we hypothesize that engorged nymphs arriving on migratory birds in spring are able to moult to adults under the current Dutch climatic conditions in spring and summer. *H. marginatum* is currently not able to establish permanent populations due to the climate. For *H. rufipes*, which is distributed in drier areas of Africa and prefers arid conditions [35], it seems even more unlikely that permanent populations will be established in the Netherlands, which is classified as Cfb (temperate oceanic) according to the Köppen–Geiger climate classification [57].

CCHFv was not detected in any of the tested ticks (13 adults and one nymph). False-negative results for CCHFv (a RNA virus), due to the time it took to arrive at the laboratory after discovery (average 1.9 days, range 0–4 days) or issues of storage by citizens and transport, obviously cannot be ruled out. However, our findings are in accordance with other studies performed in western and northern countries which tested for CCHFv in *Hyalomma* ticks [11, 16–18, 20, 22, 23]. CCHFv-positive *Hyalomma* ticks can be found in ticks collected in regions in and around Europe that lie closer to CCHFv endemic regions [8, 9, 58]. CCHFv also seems to have a focal geographic distribution range in regions where competent vector tick species are established [33]. Complex interactions between tick vector species, reservoir hosts, climate (change) and social changes leading to alterations in vegetation and use of landscape determine the enzootic cycle of CCHFv and human outbreaks [59]. Widespread occurrence of vectors and reservoirs does not necessarily lead to outbreaks of CCHFv in humans, and even though climate may favour competent tick populations, it is not directly linked to the presence of CCHFv [60]. This makes it difficult to predict the dynamics of CCHFv in areas where potential reservoirs are present, the competent vectors are occasionally introduced and climate is changing, as is the current situation in the Netherlands. CCHFv was recently found in *Hyalomma* ticks in Spain [61], and at a later stage evidence of circulation of the virus in wildlife was found in parts of Spain, including human cases [62, 63]. The virus strain found in Spain belongs to a cluster of strains isolated in western Africa, and it was estimated that it was introduced in Spain around 50 years prior to the first human cases [64]. This finding shows that enzootic cycles can be present unnoticed for quite some time. Although other tick species are also involved in the epidemiology of CCHFv, *H. marginatum* is considered the principal vector in Europe [33, 65]. Because both transstadial and transovarial transmission of the virus in ticks is possible, *Hyalomma* ticks can also form a reservoir of CCHFv [53]. To estimate the risk of introduction of CCHFv via *Hyalomma* ticks imported by migratory birds, the dynamics between the virus, the tick and feeding on a non-viremic host need to be clarified. In summary, taking into account that (i) only a small percentage of larvae in endemic areas are infected via transovarial transmission [45, 60, 66], (ii) viremic transmission of ticks via migratory birds [60, 67] and transmission of CCHFv via co-feeding on migratory birds seems unlikely [33], (iii) transstadial transmission is only successful in a little more than a third of the ticks and perhaps lower in ticks feeding on birds [66, 68], (iv) *H. marginatum* immatures are most likely dropped off only occasionally in northern and western European regions, and (v) no CCHFv has been found so far in *Hyalomma* ticks in northern and western European countries, the chance of human exposure to CCHFv via *Hyalomma* ticks in the Netherlands is currently considered very low.

The presence of the strictly intracellular bacterium *Rickettsia,* of which the species *aeschlimannii* was found in our study in one tick, is reported much more often in *Hyalomma* ticks [14, 17–19, 23, 51, 58, 69, 70]. The reason for this might be that *Rickettsia* spp. are considered heritable symbionts from invertebrates [71, 72].

The ticks tested negative for the parasites *Babesia* and *Theileria*. This is in accordance with other studies that tested *Hyalomma* ticks for *Babesia* and *Theileria* species in northern and western European countries [17, 18, 20]. Bovine tropical theileriosis (*T. annulata*) is present only in the southern parts of Europe and to our knowledge has not been detected in north-western European countries, but equine piroplasmosis (*B. caballi*/*T. equi*) is occasionally diagnosed outside its endemic range [73]. In fact, a study revealed piroplasmosis in horses in the south-western part of the Netherlands in 2010. Not all horses had been abroad, suggesting autochthonous infections, most likely caused by *Dermacentor reticulatus* ticks [74, 75]. Because *Theileria* parasites are not transmitted transovarially, the risk of the introduction of *Theileria* with *Hyalomma* ticks seems negligibly low [32]. We also estimate the risk of introducing *Babesia* through *Hyalomma* ticks as very low, although it must be taken into account that this protozoan can be transmitted transovarially [76].

## Conclusion

*Hyalomma* ticks are regularly introduced into areas outside their current living range, and our results show that alert citizens can detect the presence of a *Hyalomma* tick and report the sighting to the authorities. We hypothesize that as of summer 2019, citizens were more alert to *Hyalomma* ticks than in previous years. The awareness, caused by media reports of sightings, in combination with the intensive care (e.g. brushing) of horses could be the reasons that a relatively large number of ticks were reported in 2019 and 2020 compared to previous years, and that most *Hyalomma* ticks were found on horses. Under our current climatic conditions, introduced engorged nymphs are able to moult into adults, but the establishment of permanent *Hyalomma* populations is considered unlikely. We estimate the current probability of introduction of *Hyalomma* tick-borne pathogens in the Netherlands as very low, with the exception of *R. aeschlimannii*, of which there is existing risk, as demonstrated by our results, and to a lesser extent *B. caballi*.

## Supplementary Information

**Additional file 1**: **Table S1**. Graphic image of all reports to the CMV, including *Hyalomma* (June 2019–December 2020). **Table S2**. *Hyalomma* sp. GenBank^®^ accession numbers (*n*=77) used for the cluster analysis.

## Data Availability

*Hyalomma* sequences generated in this study were submitted to NCBI GenBank^®^ under accession numbers MT757612 to MT757622, and MW495246 to MW495248. The *Rickettsia aeschlimannii* sequence generated in this study was submitted to the NCBI GenBank^®^ under accession number MW498244. Data supporting the conclusions of this article are included within the article and its additional file. A limited amount of DNA/RNA from samples is available upon reasonable request.

## Abbreviations

CCHFv: Crimean–Congo haemorrhagic fever virus
CMV: Centre for Monitoring of Vectors
gltA: Citrate synthase gene
NVWA: Netherlands Food and Consumer Product Safety Authority
ompB: Outer membrane protein B gene
RIVM: National Institute for Public Health and the Environment
s.l.: Sensu lato
UPGMA: Unweighted pair group method with arithmetic mean
